# Adverse events and contradictory effects of benzodiazepine in a case with intellectual disability and challenging behaviour

**DOI:** 10.1177/17446295221134420

**Published:** 2022-10-25

**Authors:** Ulrich Elbing, Sebastian Appelbaum, Thomas Ostermann

**Affiliations:** 12263 Witten/Herdecke University, Alfred-Herrhausen-Straße 50, Witten 58448, Germany; 12263 Witten/Herdecke University, Alfred-Herrhausen-Straße 50, Witten 58448, Germany; 12263 Witten/Herdecke University, Alfred-Herrhausen-Straße 50, Witten 58448, Germany

**Keywords:** benzodiazepine, challenging behaviour, contradictory medication effects, intellectual disability, Markov chain

## Abstract

This study aims to contribute to the knowledge about lacking or contradictory effects of benzodiazepine on hyperaroused or aggressive behaviour in persons with intellectual disability (ID). We conducted a retrospective and natural case study in a person with mild ID and multiple adverse childhood experiences (ACE), using routine diary data consisting of 275 days including 113 consecutive days under benzodiazepine medication. The medication effects were documented as “calm / relaxed”, “fretful / distressed” and “sleep / doze”. Transitions between these were modelled using Markov chains. Differences in transitions were analysed using Chi-Square test for homogeneity. The results show a significantly reduced stability of mood and increased distressed behaviour under benzodiazepine. This is in line with reports about the effects of psychotropic medication in patients with ID and challenging behaviour. Besides other influences on unexpected medication effects, a possible dissociative identity disorder is discussed as an additional explanation.

## Background

The appropriate use of psychotropic medication in persons with intellectual disability (ID) is a topic of ongoing discussion (Bowring et al., 2017; Matson et al., 2000) including reports about substantial rates and adverse events concerning health and behavioural problems (Valicenti-McDermott and Demb, 2006), especially in benzodiazepines and their derivates (Kalachnik et al., 2002). Additionally, studies report quality problems in the documentation and administration of psychotropic medication (Deb et al., 2015; Doan et al., 2014; Lunsky et al., 2018) or inconsistent screening for side effects (Paton et al., 2016). A current review about the use of medication in adults with ID concluded that persons with ID “are at risk of medication-related harm relating to appropriateness and safety of medicine use. […] In particular, the use of multiple psychotropic agents should be frequently evaluated to assess benefits and risks.” (O’Dwyer et al., 2018). The gap between the available research literature and clinical practice seems to be ongoing, “in spite of pleas by mental health and medical disciplines to follow rules of evidence based practice” (Matson and Neal, 2009). Research concerning prescription as and when required (PRN) underlines this complaint (Delafon et al., 2013).

Only a few studies specifically deal with undesirable effects of benzodiazepines in adults with ID suggesting these are significant and troublesome. A Pubmed search in March 2020 using the search syntax “Subheading: adverse effects; MeSH terms: benzodiazepines; intellectual disability” resulted in four studies for the past 10 years after excluding the treatment of Lennox-Gastault or neuroleptic-induced akathisia and short sedations e.g. during dental surgery.

Amongst other psychotropic medication, Lunsky and coworkers (Lunsky et al., 2018) report the use of benzodiazepines in over 40.6% of 20,316 persons with ID within 2 years and stress the large proportion of psychotropic medication in general being prescribed without a psychiatric diagnosis. Scheifes and coworkers (Scheifes et al., 2016) report the use of psychotropic medication and adverse events in 103 adults with ID and very challenging behaviour; benzodiazepines being used in 36.9%. A case report (Samuel et al., 2013) describes adverse events when switching from the original to a generic drug. Another case report (Herzig et al., 2018) presents a differentiated case with polypharmacy and adverse events, including those when benzodiazepine was administered. This report shows the closest proximity to the case report presented here.

The aim of this case report is to contribute to the knowledge about lacking or paradoxical effects of benzodiazepine on hyperaroused or aggressive behaviour in persons with ID using daily diary data of a patient with ID and a history of multiple adverse childhood experiences (ACE; Brown et al., 2009; Keesler, 2014).

## Methods

This study did not require an ethical board approval because the data of this study were analysed retrospectively using routine diary notes as the only data source. There was no intervention planned or carried out in the context of this study, and no additional data were collected. Both the legal guardian of the client and the residential institution manager gave written informed consent statements concerning the publication of the diary data analysis in this paper.

### Case

“Paul” is a 54-year-old resident of a Group Home in Germany. During his first four years, Paul suffered from at least 6 out of 8 ACEs. Paul experienced frequent violent behaviour, abuse, exploitation and neglect. Psychiatric abnormalities of Paul started in preschool age with animal cruelty and tantrums or epileptic attacks. After several stays in psychiatry, Paul lived in institutions since his 7^th^ year. Reaching adulthood and after repeatedly hurting staff members severely, ongoing fixation was legally ordered. Since the age of 28 he has been living constantly in his current residential living group. In 1990, medical reports include massively aggressive, unchangeable behaviour and a summary of psychiatric findings with the diagnosis of mental disability due to early brain damage of unclear origin and a pronounced milieu damage (ICD-10 F70.1), which was considered to be the cause of his behaviour. However, due to his condition, he never had a regular psychiatric examination or diagnostic testing. A huge medication history lists various unsuccessful combinations and doses of neuroleptics, ataractics, antidepressants, lithium, antiepileptics and depot neuroleptics. Intensive therapeutic intervention was implemented in Paul's 31th year. The focus was on attachment-oriented communication and relationship in everyday life with intensive staff training and supervision. Paul then was able to move with his residential group into an external housing facility with a specially designed single room for him (Elbing, 2002).

Today, Paul is still fixed on his hands and feet every day due to his aggressive outbursts. The daily fixation-free times and the range of motion could be expanded considerably over the years. He helps actively to arrange the timing and the procedure for fixation and release from the fixation.

After a fracture of the femur with surgical intervention, there was a high risk of wounds being reopened by Paul with subsequent infection of the surgical site. Thus, the aim was to support and ensure wound healing until the forming of sustainable scar tissue. Therefore, pain management was initiated using Metamizole (500 mg tablet) five times a day, and benzodiazepine as tranquilliser (orodispersible tablet 1 mg) three times a day with no additional PRN medication. Paul stayed in hospital for 2 nights. To protect his wound, the fixation beginning with the surgery was opened for 21 days only by his personal assistants. The ongoing long-term medication stayed unchanged and consisted of antipsychotics, atypical neuroleptics and an anticonvulsant.

### Research approach

This case report is a retrospective analysis based on medical records and structured diary data. The accident mentioned above, followed by surgery and subsequently prescribed medication with Metamizole for pain relief and a benzodiazepine as a tranquillizer over a defined period of time are the basis of this case report.

### Process of Data extraction

Paul’s diary data were extracted from the institution’s diary software on a daily basis before and including 2 days in hospital following the accident (T_1_), within the medication period back home (T_2_) and after the medication period (T_3_). Paul’s mood was routinely recorded on a daily basis covering morning, noon, afternoon and night. The notation was done by staff members at defined time points of shift change, covering the behaviour during the last shift. The notation was double checked by Paul’s personal assistant on duty (four eyes principle of control). It covered three categories: A: calm/relaxed, B: fretful/distressed, C: sleep/doze based on the window of tolerance model of arousal regulation (Siegel, 1999; Corrigan et al., 2011). The category A encompasses all behaviours associated with a medium level of arousal, good psychomotor coordination, muscle tone appropriate to activity or situation and appropriate interaction with people and objects. Example: Dialogue with the staff with inconspicuous voice pressure, normal language use and adequate responsiveness to the previous conversation. Regular sleep at night is also coded with A. Category B includes all behaviours associated with a significantly increased level of arousal and muscle tone, reduced psychomotor coordination, limited interaction with people and objects and expansive verbal and physical behaviour. This encompasses behaviours from making demands in an aggressive voice, to very loud insults in fecal language, to assault and property damage with and without loss of control. Category C includes all behaviours associated with decreased arousal level, flabby muscle tone, slowed and/or delayed response, weak movements, slowed-down, slurred speech to salivation and inactivity until sleep at day time.

### Hypotheses

With respect to the initiated pain and sedation management in T_2_ it was firstly supposed that Paul’s mood was stable with the stage “sleep/doze” being a recurrent stage with less transitions into other stages. As a consequence it was supposed that transition probabilities behave differently in the medication period compared to the baseline period T_1_ and the period after surgical intervention T_3_. Thirdly, we assumed no differences in transition probabilities between the stages in T_1_ and T_3_.

### Markov Chain approach

A Markov chain model based on the three stages A, B and C was set up and transition probabilities between the stages were calculated separately for the three time frames based on the treatment diary data. Using these transition probabilities, the Markov property claiming that the future behaviour of the chain only depends on its present value and not on its past behaviour was analysed using the R package “markovchain” (Spedicato, 2017). Using a Chi-Square Test for Homogeneity (Anderson and Goodman, 1957) we tested differences in transitions between the states between the time frames T_1_, T_2_ and T_3_. Moreover, we also tested whether the Markov chains were irreducible and which states were transient, recurrent or absorbing. The level of statistical significance was defined as α = 0.05.

## Results

78 days (including 2 days in hospital) in T_1_, 113 days in T_2_ and 84 days in T_3_ provided sufficient diary data to calculate the transition probabilities. Figure one provides the percentages of stages in the three time periods. Corresponding to the sedating and tranquilizing medication, the stage C “sleep/doze” in total has increased from 2.3% in T_1_ to 10.9% in T_2_ and the falls back to 2.1% in T_3_. Especially stage B “fretful/distressed” showed a significant increase in T_2_ compared with T_1_ and T_3_ (Chi-Square: 98.92, df = 4, p < 0.0001).

**Figure 1.**
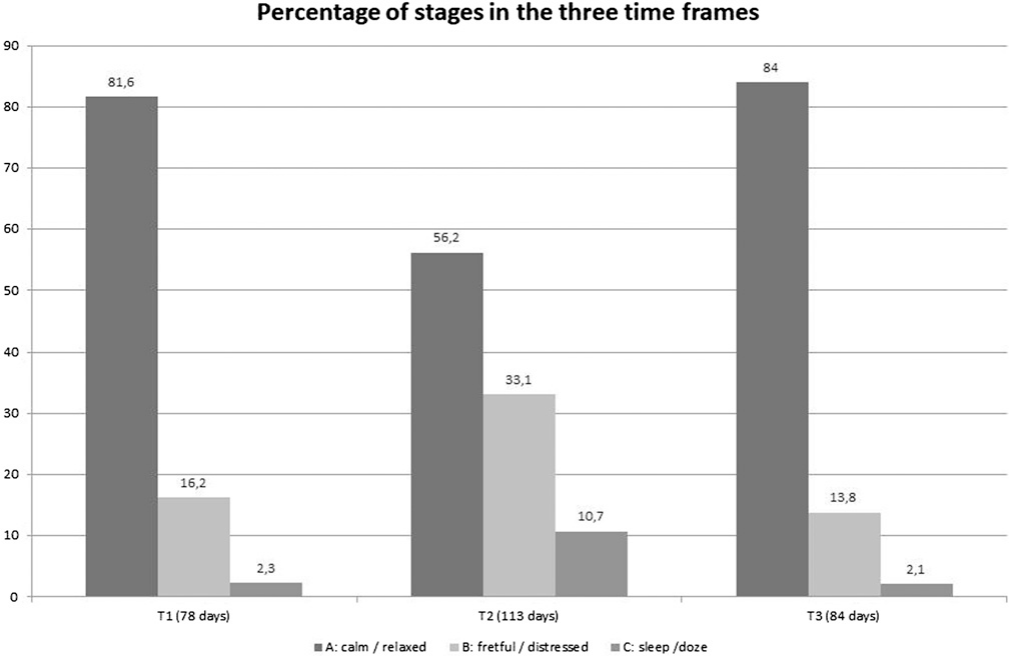
Percentage of stages A: calm/relaxed, B: fretful/distressed, C: sleep/doze in the three time frames.

**Figure 2.**
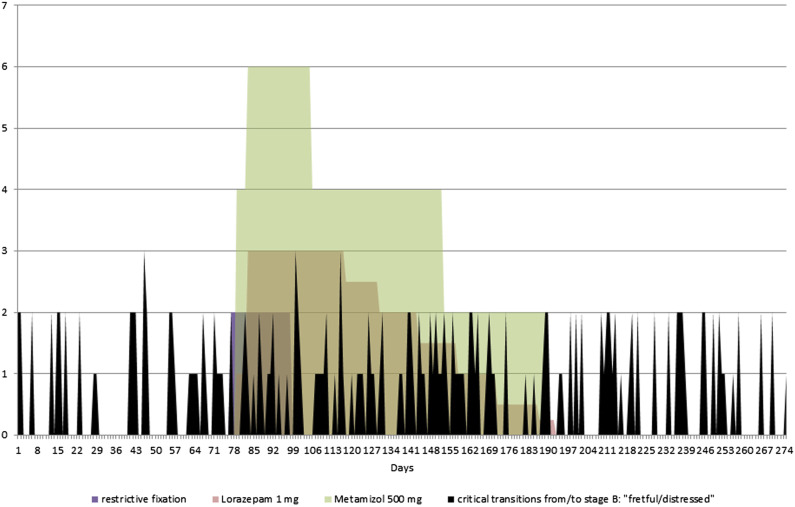
Time line of critical transitions from/to stage B: “fretful/distressed”.

The observed critical transitions to or from the stage of being “fretful/distressed” (stage B) were documented for the whole time line in Fig. 2, displaying not only an increase in the number of stage B: “fretful/distressed” as in Fig. 1, but an increase of critical transitions during the medication period as well.

**Figure 3.**
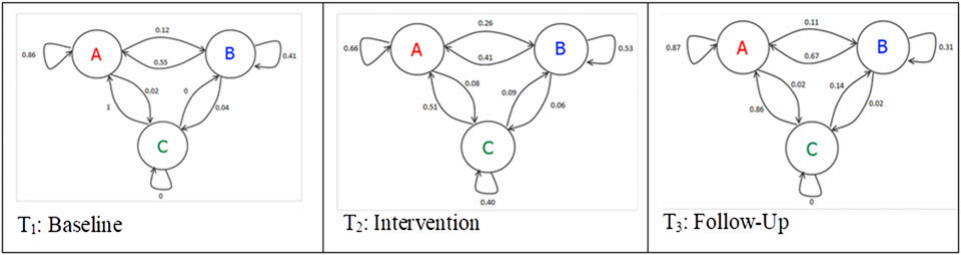
Markov chain for the three time frames T_1_ Baseline, T_2_ Intervention and T_3_ Follow-Up with stages A: calm/relaxed, B: fretful/distressed, C: sleep/doze and transition probabilities in the three time frames.

Transition probabilities between the stages for each period are provided in Fig. 3. Test for Markov property was positive for each time frame. Corresponding to the different distribution of stages in Fig. 1, Chi-Square test for homogeneity revealed differences in transitions between the time frames T_1_ and T_3_ compared with T_2_ (T_1_ vs. T_2_: Chi-Square: 100.494, df = 8, p < 0.0001; T_3_ vs. T_2_: Chi-Square: 124.741, df = 8, p < 0.0001) while there was no difference between T_1_ and T_3_ (Chi-Square: 3.792, df = 8, p = 0.875).In all time periods the Markov chain was irreducible and all stages were recurrent.

Category B and C conditions occurred significantly more frequently in period T_2_ than in periods T_1_ and T_3_. Moreover, critical transitions to and from state B occurred significantly more frequently in the period T_2_.

## Discussion

The case report presented here offers the opportunity to investigate the effects of a changed setting including the loss of function and impairment, the change in environment i.e. admission to hospital and notably the prescription of benzodiazepine. Our findings suggest an increase in hyperaroused, distressed and aggressive behaviour during the period that benzodiazepine was used. Additionally, the findings suggest an increase of behavioural changes as well, indicating a higher fluctuation of behaviour in this period.

### Weaknesses of the data and results

The case presented here is associated with several methodological restrictions which have to be accounted for when interpreting the results. Because the report is retrospective, the basis of the evaluation inevitably consists of already available data with all its methodological weaknesses. Thus, there was no possibility of blinding when administering medication, testing the reliability of the documentation and performing initial diagnostics according to current scientific standards. Although the four-eyes principle of mutual control supports quality assurance, it does not ensure reliability. However, as a longer observation period allows for the expectation of a levelling of the observation with increasing routine, significant differences over time would rather be expected with shorter time series. Highly significant differences despite the length of the time series as demonstrated in our case could nevertheless indicate a sufficient reliability.

Furthermore, the differences found can be attributed to an observer bias, which is due to both the behavioural expectations after the surgery and the expected effects of the additional medication. This bias certainly affects the interpretability of the differences found. On the other hand, the long period of 113 days until the additional medication is completely withdrawn may counteract the maintenance of the expectations mentioned above.

### Possible explanations for the results found

Assuming that the documented changes in T_2_ are based not only on observation errors, but also on actual changes in behaviour, they can be plausibly explained by the accompanying circumstances and consequences of the accident: psychological effects of the accident, hospital experience and the subsequent narrow fixation regime; pain; effects of restricted movement in everyday life; reaction to the fear of the employees of more frequent or severe aggressive outbreaks etc. These circumstances are likely to increase arousal, anxiety and fear, and the benzodiazepine may have additionally contributed to the volatiliy of behavioural changes by inhibiting the inhibitory neurotransmission, thus failing to balance the exictatory neurotransmission. A possible assessment of the pain medication as less suitable for effectively preventing the pain might also explain the increased instability and restlessness. Likewise, possible withdrawal symptoms when stopping the benzodiazepine medication can explain increased instability and restlessness. In this case, the step-by-step procedure would not have been sufficient. However, the data show no substantial differences in the critical transitions; the time of the initial administration of 3 x 1 mg benzodiazepine over 24 days is certainly not characterised by significantly less instability and restlessness (see Figure 2) than the withdrawal phase.

Benzodiazepine was in fact prescribed to avoid behavioural consequences of the accident. Its regular administration intended to achieve reliable sedation which was not successful. In addition, with an orodispersible administered benzodiazepine, a clear effect is normally expected within minutes. This improves its observability compared to psychotropic drugs with a slower onset of effect. The increased frequency of overexcited behaviour found when administering benzodiazepine, on the other hand, contributes to the findings on adverse events (Scheifes et al., 2016). In particular, the finding of more frequent critical changes under medication deserves attention because benzodiazepine was administered not as a PRN but as a continuous medication, regardless of the respective behaviour. The explanation of a paradoxical effect in the case of hyperarousal (which would correspond to a dose as PRN) does not apply in this case. Neurophysiological peculiarities in the presence of brain damage can fundamentally explain inter-individually different reactions to psychotropic medication compared to the general population (O’Dwyer et al., 2018). In our opinion, more frequent and less predictable intra-individual fluctuations in behaviour under medication are not exhaustively explained by a history of brain damage as a constant condition. A possible additional explanation can be provided by the discussion on the findings of intra-individual effects of psychotropic drugs that are difficult to predict in people with dissociative identity disorder (International Society for the Study of Trauma and Dissociation, 2011). The critical accumulation of adverse childhood experiences suggest that this explanation may reflect a relevant aspect of Paul's behavioural fluctuations under medication.

## Conclusion

The phenomenon of fluctuating intra-individual predictability of psychotropic medication effects is underpinned by our findings. Further research with individual long term case studies will be required to prove and describe this phenomenon of intra-individual fluctuations in effects in more detail.

Besides the findings presented, this case study is one more call for the need for ongoing routine documentation that is capable of mapping the effects of psychotropic medication. Despite all the limitations (see above), the documentation presented is sufficiently suitable to depict changes in a way that it could have been used to control and adapt psychotropic medication timely and effectively. However, the documentation obviously was not used in this way. On the basis of the documentation, however, an alarm could have been set up with little additional effort, which timely indicates the lack of reasonable effect. Therefore, this study additionally stresses the need to combine documentation with a timely alarm routine to adapt or correct the prescription of psychotropic medication. Given the prescription practice for psychotropic medication for people with ID (O’Dwyer et al., 2018) this is necessary not only in hospitals, but in residential institutions as well.
